# Factors Associated with Poor Glycemic Control Amongst Rural Residents with Diabetes in Korea

**DOI:** 10.3390/healthcare9040391

**Published:** 2021-04-01

**Authors:** Junhee Ahn, Youngran Yang

**Affiliations:** 1 Department of Nursing, Kunjang University, Gunsan-si 54045, Korea; acsac@hanmail.net; 2 College of Nursing, Research Institute of Nursing Science, Jeonbuk University, Jeonju-si 54896, Korea

**Keywords:** rural residents, diabetes, Hemoglobin A1c, poor glycemic control

## Abstract

(1) Background: Glycemic control is an effective way to reduce the cardiovascular complications of diabetes. The purpose of this study was to identify the factors associated with poor glycemic control amongst rural residents with diabetes in Korea. (2) Methods: This cross-sectional analysis was conducted amongst a total of 522 participants who had completed baseline health examinations for the Korean Genome and Epidemiology Study (KoGES) Rural Cohort from 2005 to 2011. The subjects were divided into two groups: the good glycemic control group (GCG) (glycosylated hemoglobin (HbA1C) < 7%) and the poor GCG (HbA1C ≥ 7%). Logistic regression was used to examine the role of sociodemographics, health-related behavior, comorbidity and diabetes-related and clinical factors in poor glycemic control amongst rural residents with diabetes. (3) Results: In total, 48.1% of participants were in the poor GCG. Poor GCG was significantly associated with drinking (odds ratio (OR) = 0.42, 95% CI = 0.24–0.71), lack of regular physical activity (OR = 1.68, 95% CI = 1.03–2.76), fasting blood glucose (FBG) > 130 mg/dL (OR = 7.80, 95% CI = 4.35–13.98), diabetes for > 7 years (OR = 1.79, 95% CI = 1.08–2.98), cholesterol ≥ 200 mg/dL (OR = 1.73, 95% CI = 1.05–2.84) and positive urine glucose (OR = 6.24, 95% CI = 1.32–29.44). (4) Conclusion: Intensive glucose control interventions should target individuals amongst rural residents with diabetes who do not engage in regular physical activity, have been diagnosed with diabetes for more than seven years and who have high fasting-blood glucose, high cholesterol levels and glucose-positive urine.

## 1. Introduction

Type 2 diabetes is a lifestyle-related disease and is gradually increasing due to inactivity, sedentary lifestyles, obesity and Westernised food intake. The number of people with diabetes worldwide has increased more than fourfold in 35 years, from 118 million people in 1980 to 422 million in 2014. In 2016, diabetes was estimated to be the seventh leading cause of death globally [1]. Diabetes increases the risk of several complications, such as myocardial infarction, stroke, kidney failure, lower extremity amputation and loss of vision if blood sugar levels are uncontrolled for long periods of time, as well as premature death [2]. The prevalence of diabetes in Korea is also increasing; 13.8% of adults over 30 have diabetes [3], and associated diseases, such as hypertension, liver disease and kidney disease, appear in 88% of Koreans with diabetes [4]. As these complications emerge gradually and without symptoms that patients are likely to experience in their daily lives, self-management measures, such as steady medication, healthy eating, exercise, blood glucose measurement and smoking cessation, are important for effective blood glucose management and maintenance [5,6]. Glycemic control is the most effective means of preventing complications associated with diabetes. However, a ratio that is controlled to under 7% of HgbA1C—the ideal blood sugar control target for type 2 diabetes patients—is very low, at 35% in the United States [7], 37% in Europe [8], 47% in Canada [9] and 70% in Korea [3], meaning that 53~70% of diabetic patients with uncontrolled blood glucose levels require thorough self-care, external intervention and help to prevent complications and reduce mortality.

Local socioeconomic levels affect the health of individuals living in the area, and social factors such as the community’s material resources play a decisive role in maintaining health-related behavior [10]. Korean society promotes rapid industrialization, which creates environmental gaps between urban and rural areas [11]. Consequently, rural residents often face barriers to health care, which may exacerbate these disparities. Medical institutions and providers are concentrated in metropolitan areas, leading to health inequality due to gaps in the accessibility of medical institutions and the regional quality of medical services [12]. In 2018, the prevalence of diabetes in Korea was 11.8% in urban areas and 15.8% in rural areas [13]; the adequate blood glucose control rate was 50% in Seoul and 36.5% in the Chungcheong province, highlighting the regional gap [14]. Limited options and access to medical institutions and high medical expenses associated with diabetes treatment, in tandem with lower income, lack of diabetic specialists and fewer opportunities for diabetes education focusing on self-management in rural areas, likely account for these inter-regional differences [15]. Rural areas are located in a blind spot with respect to economic, medical and social services in comparison to cities [16,17] and require a targeted blood glucose management care plan.

Some notable studies around the world have identified the following: education up to high school level only, residency in regions outside of Seoul, long-term diabetes, combined insulin and oral hypoglycemic treatment, hypertriglyceridemia, excessive sleep, hypertension, smoking and weight fluctuation were identified as contributing factors in uncontrolled blood glucose in Korean diabetic patients [14,18,19]. A Yemeni study showed that 73.2% of diabetic outpatients did not take measures to control their blood glucose, particularly women [20]. A younger age, lower education level, lack of regular exercise, a diabetes diagnosis of more than seven years, insulin treatment and albuminuria have been shown to cause uncontrolled blood glucose levels. Rural residency, low education level, diabetes prevalence for over 10 years and working in sales were factors of poor glycemic control amongst Ethiopian diabetes patients [21].

As such, several differences between countries are evident in the factors contributing to uncontrolled blood glucose in rural areas, but studies on blood glucose control that reflect the medical accessibility, environment and specificity of rural areas have been very limited. Due to the recent ageing of the global population, the prevalence of diabetes in rural areas has increased significantly; consequently, it may become more important for diabetic patients to control their blood glucose levels to prevent diabetes complications, save on medical expenses and improve their quality of life. Therefore, this study investigated the status of glycemic control and the demographic, lifestyle, diabetes-related and clinical factors associated with poor glycemic control amongst diabetic patients in rural areas of Korea. This study’s findings will guide the development of interventions aimed at preventing complications and reducing medical expenses and mortality in rural residents with diabetes.

## 2. Materials and Methods

### 2.1. Study Participants and Data Collection

This study used secondary data from the Rural Cohort of the Korean Genome and Epidemiology Study (KoGES) to identify the factors affecting uncontrolled glycemic status in rural diabetic patients. KoGES has established a large-scale cohort for the prevention and management of cardiovascular disease for the general population aged between 40 and 69 years by the Korea Disease Control and Prevention Agency (KDCA). In this study, we used the baseline survey data of men and women aged between 40 and 69 years in six rural areas—Yangpyeong, Namwon, Goryeong, Wonju, Pyeongchang and Ganghwa—from 2005 to 2011. In compliance with KoGES’s confidentiality protection regulations and statistical laws, the KCDC provides only non-identifiable data. The data were obtained with the approval of Jeonbuk National University’s Institutional Review Board (IRB No. 2019-10-010).

Of the 28,337 people who participated in the baseline survey of the Rural Cohort, 2656 diabetic patients answered “Yes” to the question of “diabetes diagnosis” according to an earlier study [22]. After omitting 2134 people for whom HbA1C data were missing, 522 subjects’ data were used for the final analysis.

### 2.2. Definition of Poor Glycemic Control

Participants with less than 7% HbA1C were classified as the “good glycemic control group” while those with over 7% were categorized as the “poor glycemic control group”, with the aim of reducing microvascular and macrovascular complications in diabetic patients according to the guidelines of the American Diabetes Association (2019) [5]. In KoGES, HbA1C was measured using high-performance liquid chromatography (Variant II TURBO; BioRad, Hercules, CA, USA) in accordance with the National Glycohemoglobin Standardization Program.

### 2.3. Measurements of Related Factors

Variables related to glycemic control were classified into demographic, health-related behavior, disease-related, diabetes-related and clinical examination factors by selecting available variables from KoGES data based on earlier studies. General characteristics were grouped as follows: age (40–64 years old and 65 years or older), marital status (currently married, unmarried, divorced, separated and widowed), education level (primary, middle, high school and above), monthly household income (less than 1 million won, 1 million to 1.99 million won and 2 million won and above; in consideration of economic conditions in rural areas).

Health-related behavior and disease-related factors were included and classified as follows [19]: drinking (non-drinking, current drinking), smoking (non-smoking, current smoking), regular physical activity (responses of yes/no to the question “Do you regularly exercise enough to produce sweat?”). Comorbid diseases, including hypertension, hypertriglyceridemia and stroke diagnosis, were classified as self-reported in the health survey. As diabetes-related factors, the duration of diabetes (more or less than seven years) based on earlier studies [18,20] and use of drug treatment (oral hypoglycemic agent or insulin injection) and non-drug treatment methods (diet or exercise) were included. Cholesterol, triglyceride (TG), high-density lipoprotein cholesterol (HDL-C), fasting blood glucose (FBG), low-density lipoprotein cholesterol (LDL-C), blood pressure and body mass index (BMI) were selected as clinical examination factors. The cut-off points for each were as follows: cholesterol (200 mg/dL), triglyceride (150 mg/dL), HDL-C (40 mg/dL for men, 50 mg/dL for women), FBG (130 mg/dL) and LDL-C (100 mg/dL). LDL-C was estimated using Friedewald’s formula (LDL-C = total cholesterol-(HDL-C)-(triglyceride/5)) [23]. Blood tests were performed after subjects had fasted for over eight hours (ADVIA1650, Siemens, New York, NY, USA). Proteinuria and urine glucose were divided into negative (negative and trace) and positive (1+ ~ 4+). Blood pressure was classified based on systolic pressure of 140 mmHg or more and diastolic pressure of 85 mmHg or more, and BMI was classified according to the World Health Organization (WHO) Asia Pacific guidelines into normal at less than 23 kg/m^2^, overweight between 23 to 24.9 kg/m^2^ and obese at 25 kg/m^2^ or more [24].

### 2.4. Data Analysis

Using SPSS/Win 21.0, frequency, percentages, mean and standard deviations were obtained for general characteristics and disease-related characteristics. A Chi-square test was performed to determine the differences in poor glycemic control according to demographic, health behavior, disease factors, diabetes-related and clinical examination characteristics. A binary logistic regression analysis was performed to identify factors affecting poor glycemic control.

## 3. Results

### 3.1. The General Characteristics of Participants

The participants were 522 rural diabetic patients, of whom 57.9% were women. The 40–64 years age range accounted for 68.8% and the average age was 60.2 (±7.84) years old. It was found that 82% of the participants did not live with their spouses and 50.6% of participants were educated up to the primary school graduation or less. The monthly household income of 54.7% participants was less than 1 million won and 56% were currently employed. Diabetes duration of seven years or more was found in 38.1% of subjects; 25.9% used oral hypoglycemic agents or insulin injection treatment and 2.5% used diet or exercise therapy as non-drug treatments.

### 3.2. Differences in Glycemic Control According to Participants’ Characteristics

Amongst 48.1% of rural diabetic patients, HbA1C was uncontrolled at more than 7%. Poor glycemic control rates were higher in non-drinking than drinking subjects (53.1% vs. 39.1%, *p* = 0.003); in those who did not engage in regular physical activity than those who regularly engaged in physical activity (54.5% vs. 41.4%, *p* = 0.002); in those whose diabetes duration was >7 years as opposed to ≤7 years (59% vs. 40.7%, *p* < 0.001); in those whose FBG > 130 mg/dL as opposed to 70–130 mg/dL (81.7% vs. 31.2%, *p* < 0.001); in those whose cholesterol ≥ 200 mg/dL as opposed to < 200 mg/dL (57.3% vs. 42.5%, *p* < 0.001) and in those whose urine glucose was positive rather than negative (91.1% vs. 44%, *p* < 0.001) (Table 1).

### 3.3. Factors Associated with Poor Glycemic Control

Table 2 presents the results of logistic regression analysis showing the influences of drinking habits, regular physical activity, duration of diabetes, fasting blood glucose, cholesterol and urine glucose that were found to be significant in the Chi-square test on HbA1C control. Poor glycemic control was significantly (0.42 times) lower (95% CI = 0.24–0.71) in the drinking group than in the non-drinking group; 1.68 times higher (95% CI = 1.03–2.76) amongst the group who did not engage in regular physical activity than the group who partook in physical activity; 7.80 times higher (95% CI = 4.35–13.98) amongst individuals with FBG > 130 mg/dL than those whose FBG was between 70 and 130 mg/dL; 1.79 times higher (95% CI = 1.08–2.98) amongst individuals with diabetes for >7 years as opposed to ≤7 years; 1.73 times higher (95% CI = 1.05–2.84) amongst individuals with cholesterol ≥200 mg/dL as opposed to <200 mg/dL and 6.24 times higher (95% CI = 1.32–29.44 amongst individuals with positive rather than negative urine glucose.

## 4. Discussion

Diabetes is increasing globally due to changes in ageing, diet and lifestyle, and diabetes management in rural areas is poor compared to that in cities due to the lack of medical access and medical information [25]. Several studies have identified the relevant factors in uncontrolled blood glucose [7,8,9,14,19], but studies conducted specifically amongst rural diabetic patients are lacking. This study sought to identify the demographic, health-related behavior and diabetes-related factors associated with poor glycemic control in rural diabetic patients using the Rural Cohort data of KoGES in order to provide information for the development of a management program and customized intervention plans aimed at preventing diabetes-related complications.

The rate of uncontrolled blood glucose of HbA1C of 7% or higher in this study was 48.1% compared with 35% in the United States [7], 37% in Europe [8] and 47% in Canada [9], suggesting that effective and tailored intervention is necessary for controlling blood glucose levels in rural Korean diabetic patients. In a study by Park et al. (2016) [14], poor glycemic control (HbA1C 7% or higher) was found to be 1.97 times higher in the Chungcheong province and 1.74 times higher in the Jeolla/Jeju regions than in Seoul. As noted above, medical institutions and medical providers are concentrated in metropolitan areas, so local medical accessibility and quality of medical services in rural areas may be low. The related literature supports this claim. For example, according to research from Myanmar, differences are evident in the prevention and control of diabetes by region, and measures aimed at preventing diabetes in rural areas are insufficient [25,26]. This suggests that a strategy acceptable and feasible for rural residents is required to increase blood glucose control rates because differences in the systems, environments and resources between rural and urban areas impact the effective management of diabetes.

As a result of the logistic regression analysis carried out in this study, non-drinkers, those with diabetes duration > 7 years, those with FBG > 130 mg/dL, those with cholesterol ≥ 200 mg/dL and those who tested positive for urine sugar showed significantly higher poor glycemic control rates than their counterparts. Those who drank had 0.42 times lower blood glucose control than the non-drinking group. This echoes findings that drinkers have better blood glucose control than non-drinkers [14] but differs significantly from studies that have indicated that drinking status and blood glucose control are not associated [19] and that non-drinking groups show better glycemic control [27]. It is thought that this difference in research results is due to different standards and definitions for the amount, type and duration of drinking for each study. Alcohol products with many calories and without nutrients are easily absorbed by the body, leading to increased risk of diabetes, high blood glucose levels and weight gain [28,29]. In patients with hypoglycemia (sulfonylurea) and who are treated with insulin injections, drinking is a risk factor [30]. Therefore, it is important to emphasize that diabetic patients whose HbA1C remains within the target achievement range could develop hypoglycemia due to their drinking habits and to educate them as to the need to abstain from alcohol to achieve blood glucose control goals while engaging in an appropriate level of exercise. HbA1C shows the degree of blood glucose control for the last three to four months, but not the level of hypoglycemia [31]. The higher level of poor glycemic control in the non-drinking group may indicate that the drinking group became cautious about monitoring their normal blood glucose levels and started abstaining from drinking. However, the exact cause is unknown, as this study is a cross-sectional survey in which the cause–effect relationships cannot be identified. Thus, future research should include a follow-up study on the effects of alcohol consumption on uncontrolled blood glucose.

In this study, the group who did not engage in regular physical activity had a poor glycemic control rate which was 1.68 times higher than that of their more active counterparts. This echoes findings that regular exercise positively affects uncontrolled blood glucose [20] but differs from findings that have discovered no such effect [19]. Each study implements different criteria for exercise; in previous studies, for instance, strength training and regular exercise three to five days per week were used. The question in this study asked whether they exercise enough to produce sweat. Therefore, further repeated studies with standardized criteria are recommended. Ultimately, regular physical activity is widely recommended as a non-drug therapy that helps prevent complications by controlling blood glucose levels, managing weight and decreasing body fat in diabetic patients. Continuous education during the non-farming season in rural areas and the creation of exercise-friendly environments by equipping village centers with facilities, for example, are necessary for promoting regular exercise habits so that rural diabetic patients can maintain appropriate blood glucose levels.

In this study, the observed correlation between FBG levels and HbA1C was strong (7.80 times higher in poor glycemic control for FBG > 130 mg/dL than normal FBG). When HbA1C data are unavailable, FBG can substitute as a guide for blood glucose management for rural residents. However, amongst this study’s participants, 31.2% of those with normal FBG at 70–130 mg/dL had poor blood glucose conditions according to the HbA1C standard, which indicates the degree of blood sugar management in the past three to four months. Therefore, it is recommended that diabetic patients regularly check their HbA1C—at least two to four times per year—and that they are provided with access to clinics for HbA1C blood tests.

There are several ways to measure the level of glycemic control. HbA1C reflects the level of blood sugar during last 2–3 months. It can be measured regardless of fasting state, less susceptible to stress or infection than fasting blood glucose, and has little variation within individuals [32]. Urine glucose is not sensitive to glucose control because the extracellular glucose does not increase within 180 mg/dL of fasting blood glucose among most patients [33]. HbA1C can be confounded in the anemia, hemoglobinopathy, or renal impairment. In comparison, glycated albumin (GA), a ketoamine formed by binding of albumin and glucose, more accurately reflects short-term changes in plasma glucose and postprandial plasma hyperglycemia (PPH). GA is not affected by hemoglobin or dialysis [34,35]. 1,5-Anhydroglucitol (1,5-AG), another potential glycemic marker, structurally resembles glucose and decreases with spikes of hyperglycemia exceeding the average renal threshold for glucose. Unlike HbA1C, it is known that it is not affected by the state of red blood cells, intraday fluctuations, weight, age, temporary dietary changes, exercise, and acute renal failure [35]. Given these different measures of glycemic control, clinicians should use the best marker of glycemic control with consideration of the patient’s condition and medical resources available.

When the patient had diabetes for longer than seven years, the rate of unregulated blood glucose was 1.79 times higher than that of patients who were diabetic for seven years or fewer. This corroborates the findings of previous studies [14,18,19,20] that the longer the diabetes duration, the higher the rate of uncontrolled blood glucose. Longer duration of diabetes is associated with the deterioration of beta cell function, making it difficult to control blood glucose [36]. In addition, the patient’s self-management may cause their condition to deteriorate beyond the initial diagnosis [14]. Therefore, patients should be made aware that long-term diabetes will negatively affect their ability to control their blood glucose levels, and the obstacles to blood glucose management for patients with long-term diabetes should be identified. A study of elderly rural diabetic patients in Korea revealed that 31.3% had never received diabetes education and 29.5% reported that their lack of knowledge regarding the management of blood glucose presented a challenge to their diabetes management [37]. With the support of the government and local officials, diabetes classes should be offered in public health centers or healthcare instructors could visit villages to provide diabetes education in cases where access to such centers is limited.

In this study, when the cholesterol level was 200 mg/dL or higher, poor glucose control was 1.73 times higher than cases in which the cholesterol level was 200 mg/dL. According to a Diabetes Fact Sheet from 2018 [38], 35% of patients with type 2 diabetes in Korea have hypercholesterolemia, which increases the risk of cardiovascular disease. This corroborates the findings of Pyo et al., who showed that high cholesterol significantly increases poor glycemic control [39]. However, in studies by Gu [19], Park et al. [14] and Kim [18], cholesterol was not found to affect blood glucose levels. Dyslipidemia develops in diabetic patients due to increased fatty acid release from fat cells because of insulin resistance and increased production and secretion of low-density lipoprotein (LDL-C) and triglyceride (TG) in the liver [40], thus becoming a risk factor of uncontrolled blood glucose [41]. High cholesterol and uncontrolled blood glucose are the leading causes of death in people with diabetes as a result of increased cerebrovascular and cardiovascular diseases due to atherosclerosis [30]. Therefore, the reduction of total cholesterol through diet, medication and regular exercise can prevent and reduce major adverse cardiovascular events (myocardial infarction, stroke and cardiovascular disease) [30]. Therefore, intervention programs that combine education about how to manage blood glucose in daily life, smoking cessation, exercise and diet should be provided in addition to cholesterol management guidance for patients with cholesterol of 200 mg/dL or more in rural areas.

In this study, no significant relationship was observed between triglyceride, HDL-C, LDL-C and uncontrolled blood glucose. However, triglycerides were 170.43 ± 14.90 mg/dL on average, which was higher than 150 mg/dL, the standard for diabetic patients. Insulin resistance in diabetic patients leads to increased fatty acid release from adipocytes and increased liver LDL and Triglycemia production and secretion, leading to hyperlipidemia [5]. In this study, triglycerides and glycemic control were not significant, which was similar to the results of previous studies from Korea [19], Yemen [20], and China [42]. Since the participants of this study were not accompanied by complications of dyslipidemia, it is thought that the level of triglycerides did not rise.

The level of LDL-C was high on average, at 118.73 ± 34.08 mg/dL, which was higher than the normal standard of 100 mg/dL, and the HDL-C results were also out of normal range for both men and women. It is important to manage blood lipid levels in people with diabetes, and comprehensive intervention programs should be initiated to provide education on the causes and outcomes of hyperlipidemia in addition to education on the importance of blood glucose management.

Participants who tested positive for urine glucose had a 6.24-fold higher rate of poor glycemic control than participants with negative urine glucose. The presence of glucose in urine indicates that the blood consistently contains high levels of glucose, with the exception of glucose reabsorption due to dysfunction of the ureter [43]. Urinary glucose is a feasible approach that can be used to quantitatively and qualitatively predict diabetes by showing 80.2% sensitivity and 85.6% specificity for diabetes screening [31]. Those who have difficulty in measuring blood glycemia due to lack of access to health care institutions may be able to use it as an alternative method of determining the degree of blood glucose control by checking for the presence or absence of glucose in urine at home. According to the results of this study, 91% of diabetic patients with positive urine glucose have HbA1C of 7% or more. Thus, counselling and intervention should be provided following assessment of blood glucose control in diabetic patients according to the results of a simple urine glucose test when blood tests are inaccessible in rural settings.

Care managers (e.g., trained nurses) may take on an important role in the management of patients with diabetes by providing necessary information and helping the patients to maintain a healthy lifestyle, enhance self-efficacy to control blood glucose and achieve good compliance with their healthcare provider’s recommendations [44]. In the Korean setting, visiting nurses may play a role as care managers supported by public health assistance service programs.

The limitations of this study are as follows: First, this study has limitations in its ability to explain the causal relationship of factors affecting poor blood glucose control because of its characteristics as a cross-sectional study. There is an issue of cause versus results of glycemic control. For example, lack of physical activity can be a cause of poor glycemic control and albuminuria can be its result. Further cohort studies with different designs could overcome this limitation. Second, the diabetes diagnosis and income levels are self-reported, so their accuracy may be limited. Third, due to the limitations of secondary data, psychosocial variables, such as depression and stress, diabetes knowledge, access to healthcare service, barriers and self-efficacy, were not controlled. Fourth, the dataset used in this study does not include information on the kinds of OHA (e.g., sulfonylureas, sodium-glucose co-transporter-2 (SGL T2s). However, this study makes a meaningful contribution in comprehensively identifying the demographic, lifestyle, diabetes-related and clinical factors affecting blood glucose control in rural residents with diabetes using large-scale secondary survey data provided by the country.

## 5. Conclusions

This study analyzed the factors of poor glycemic control in 522 rural diabetic patients aged over 40 years using the Rural Cohort from the Korean Genome and Epidemiology Study (KoGES) of the KCDC. The rate of poor glycemic control was found to be 48.1% and the influencing factors were identified as alcohol consumption, physical activity, FBG, duration of diabetes, cholesterol and urine glucose. It is necessary to develop a comprehensive diabetes management program suitable for rural contexts. Furthermore, future studies should aim to identify factors that influence blood glucose control in consideration of the regional environment in addition to characteristics related to demography, health-related behavior and medical and diabetes-related factors. Multi-level analysis may be useful to reflect regional contexts like population density, local income tax, financial independence and health vulnerabilities, such as the ratios of elderly and the disabled and the number of doctors.

## Figures and Tables

**Table 1:** General characteristics of the participants and differences of glycemic control according to the characteristics.

Characteristics	Categories	All (n %)	HbA1c < 7% (n, %)	HbA1c ≥ 7% (n, %)	x^2^-Value	*p*-Value
Total †		522 (100.0)	271 (51.9)	251 (48.1)		
Sociodemographic factors						
Gender	Men	220 (42.1)	124 (56.4)	96 (43.6)	3.014	0.083
	Women	302 (57.9)	147 (48.7)	155 (51.3)		
Age (year)	Mean (SD)	60.17 ± 7.84				
	40–64	359 (68.8)	182 (50.7)	177 (49.3)	0.685	0.408
	≥65	163 (31.2)	89 (54.6)	74 (45.4)		
Spouse	Yes	94 (18.0)	53 (56.4)	41 (43.6)	0.877	0.349
	No	427 (82.0)	218 (51.1)	209 (48.9)		
Education	≤Primary school	264 (50.6)	137 (51.9)	127 (48.1)	1.225	0.542
	≤Middle school	109 (20.9)	61 (56.0)	48 (44.0)		
	≥High school	149 (28.5)	73 (49.0)	76 (51.0)		
Monthly household income (10,000 Korean won)	<100	258 (54.7)	140 (54.3)	118 (45.7)	2.238	0.327
	100–199	111 (23.5)	51 (45.9)	60 (54.1)		
	≥200	103 (21.8)	55 (53.4)	48 (46.6)		
Current job	No	228 (44.0)	117 (51.3)	111 (48.7)	0.062	0.804
	Yes	290 (56.0)	152 (52.4)	138 (47.6)		
Health behavior and comorbidity factors						
Current drinking	No	343 (66.3)	161 (46.9)	182 (53.1)	9.035	0.003
	Yes	174 (33.7)	106 (60.9)	68 (39.1)		
Duration	1–5 (years)	14 (6.5)	6 (42.9)	8 (57.1)	7.332	0.197
	6–10 (years)	21 (9.7)	13 (61.9)	8 (38.1)		
	11–20 (years)	30 (13.9)	15 (50.0)	15 (50.0)		
	21–30 (years)	44 (20.4)	21 (47.7)	23 (52.3)		
	31–40 (years)	62 (28.7)	37 (59.7)	25 (40.3)		
	>40 (years)	45 (20.8)	32 (71.1)	13 (28.9)		
Current smoking	No	465 (89.1)	244 (52.5)	221 (47.5)	0.530	0.467
	Yes	57 (10.9)	27 (47.4)	30 (52.6)		
Regular physical activity	Yes	244 (46.8)	144 (59.0)	100 (41.0)	9.510	0.002
	No	277 (53.2)	126 (45.5)	151 (54.5)		
Hypertension	No	279 (53.4)	142 (50.9)	137 (49.1)	0.250	0.617
	Yes	243 (46.6)	129 (53.1)	114 (46.9)		
Hyperlipidemia	No	433 (83)	224 (51.7)	209 (48.3)	0.034	0.853
	Yes	89 (17)	47 (52.8)	42 (47.2)		
Stroke	No	499 (95.6)	262 (52.5)	237 (47.5)	1.575	0.209
	Yes	23 (4.4)	9 (39.1)	14 (60.9)		
Diabetes-related factors						
HbA1c	Mean (SD)	7.13 ± 1.31				
Diabetes duration	Mean (SD)	7.02 ± 7.24				
	≤7	317 (61.9)	188 (59.3)	129 (40.7)	16.174	<0.001
	>7	195 (38.1)	80 (41.0)	115 (59.0)		
Diabetes Tx. OHA or Insulin	Yes	131 (25.9)	68 (51.9)	63 (48.1)	0.013	0.91
	No	374 (74.1)	192 (51.3)	182 (48.7)		
Diabetes Tx. Diet or Exercise	Yes	10 (2.5)	5 (50.0)	5 (50.0)	0.019	0.891
	No	389 (97.5)	203 (52.2)	186 (47.8)		
Fasting blood glucose (mg/dL)	Mean (SD)	127.22 ± 38.15				
	70–130	346 (66.4)	238 (68.8)	108 (31.2)	118.715	<0.001
	>130	175 (33.6)	32 (18.3)	143 (81.7)		
Clinical-related factors						
BP (mmHg)	Mean (SD)	126.69 ± 17.72/75.85 ± 10.51				
	<140 or 85	365 (70.2)	193 (52.9)	172 (47.1)	0.446	0.504
	≥140 or 85	155 (29.8)	77 (49.7)	78 (50.3)		
BMI (kg/m^2^)	Mean (SD)	25.21 ± 3.17				
	<25	251 (48.1)	133 (53.0)	118 (47.0)	0.223	0.637
	≥25	271 (57.9)	138 (50.9)	133 (49.1)		
Cholesterol (mg/dL)	Mean (SD)	192.65 ± 37.45				
	<200	322 (61.8)	185 (57.5)	137 (42.5)	10.703	0.001
	≥200	199 (38.2)	85 (42.7)	114 (57.3)		
Triglycemia (mg/dL)	Mean (SD)	170.43 ± 14.90				
	<150	291 (55.9)	160 (55.0)	131 (45.0)	2.635	0.105
	≥150	230 (44.1)	110 (47.8)	120 (52.2)		
HDL-C, male (mg/dL)	Mean (SD)	39.84 ± 9.31				
	≥40	82 (37.4)	47 (57.3)	35 (42.7)	0.071	0.790
	<40	137 (62.6)	76 (55.5)	61 (44.5)		
HDL-C, female (mg/dL)	Mean (SD)	40.81 ± 8.86				
	≥50	45 (14.9)	18 (40.0)	27 (60.0)	1.593	0.207
	<50	257 (85.1)	129 (50.2)	128 (49.8)		
LDL-C (mg/dL)	Mean (SD)	118.73 ± 34.08				
	<100	147 (28.2)	80 (54.4)	67 (45.6)	0.554	0.457
	≥100	374 (78.1)	190 (50.8)	184 (49.2)		
Urine protein	Negative	478 (92.3)	254 (53.1)	224 (46.9)	3.616	0.057
	Positive	40 (7.7)	15 (37.5)	25 (62.5)		
Urine glucose	Negative	473 (91.3)	265 (56.0)	208 (44.0)	36.573	<0.001
	Positive	45 (8.7)	4 (8.9)	41 (91.1)		

Note. SD = standard deviation, OHA = oral hypoglycemic agent, BP = blood pressure, BMI = body mass index, HDL = high-density lipoprotein; LDL = low-density lipoprotein. † Totals may vary due to missing values.

**Table 2:** Factors associated with poor glycemic control.

Variable	Categories	Poor Glycemic Control n (%)	OR (95% CI)	*p*-Value
Current drinking	No	182 (53.1)	1.00	
	Yes	68 (39.1)	0.42 (0.24–0.71)	<0.001
Regular physical activity	Yes	100 (39.8)	1.00	
	No	150 (60.2)	1.68 (1.03–2.76)	0.040
Fasting glucose (mg/dL)	70–130	108 (43.0)	1.00	
	>130	143 (57.0)	7.80 (4.35–13.98)	<0.001
Diabetes duration (years)	≤7	129 (40.7)	1.00	
	>7	115 (59.0)	1.79 (1.08–2.98)	0.024
Cholesterol (mg/dL)	<200	137 (54.6)	1.00	
	≥200	114 (45.4)	1.73 (1.05–2.84)	0.031
Urine glucose	Negative	208 (83.5)	1.00	
	Positive	41 (16.5)	6.24 (1.32–29.44)	0.021

Note. OR = Odd Ratio, CI = Confidence Interval. Adjusted for diabetes treatment or management (OHA or insulin, diet or exercise) and comorbidities (hypertension, hyperlipidemia, stroke).

## Data Availability

The data that support the finds of this study are available from the corresponding author, upon reasonable request.
